# How to tackle health literacy problems in chronic kidney disease patients? A systematic review to identify promising intervention targets and strategies

**DOI:** 10.1093/ndt/gfaa273

**Published:** 2020-12-22

**Authors:** Marco D Boonstra, Sijmen A Reijneveld, Elisabeth M Foitzik, Ralf Westerhuis, Gerjan Navis, Andrea F de Winter

**Affiliations:** 1 Department of Health Sciences, University Medical Center Groningen, University of Groningen, Groningen, The Netherlands; 2 Institute for Applied Health Sciences, Coburg University of Applied Sciences and Arts, Coburg, Germany; 3 Department of Nephrology, University Medical Center Groningen, University of Groningen, Groningen, The Netherlands

**Keywords:** chronic kidney disease, health literacy, intervention, systematic review

## Abstract

**Background:**

Limited health literacy (LHL) is associated with multiple adverse health outcomes in chronic kidney disease (CKD). Interventions are needed to improve this situation, but evidence on intervention targets and strategies is lacking. This systematic review aims to identify potential targets and strategies by summarizing the evidence on: (i) patient- and system-level factors potentially mediating the relation between LHL and health outcomes; and (ii) the effectiveness of health literacy interventions customized to CKD patients.

**Methods:**

We performed a systematic review of peer-reviewed research articles in Medline, Embase and Web of Science, 2009–19. We assessed the quality of the studies and conducted a best-evidence synthesis.

**Results:**

We identified 860 publications and included 48 studies. Most studies were of low quality (*n* = 26) and focused on dialysis and transplantation (*n* = 38). We found strong evidence for an association of LHL with smoking and having a suboptimal transplantation process. Evidence was weak for associations between LHL and a variety of factors related to self-care management (*n* = 25), utilization of care (*n* = 23), patient–provider interaction (*n* = 8) and social context (*n* = 5). Six interventions were aimed at improving knowledge, decision-making and health behaviours, but evidence for their effectiveness was weak.

**Conclusions:**

Study heterogeneity, low quality and focus on kidney failure largely impede the identification of intervention targets and strategies for LHL. More and higher quality studies in earlier CKD stages are needed to unravel how LHL leads to worse health outcomes, and to identify targets and strategies to prevent disease deterioration. Healthcare organizations need to develop and evaluate efforts to support LHL patients.

**Key Learning Points**

**What is already known about this topic?**

- approximately 25% of chronic kidney disease (CKD) patients have low health literacy (LHL);
- CKD patients with LHL experience a faster disease progression and more comorbidities; and
- to improve health outcomes for CKD patients with LHL, interventions are needed, but an overview of promising intervention targets and strategies is currently lacking.

**What this study adds?**

- this systematic review has identified a variety of factors, mostly related to self-care management and utilization of care, which potentially explain why LHL patients experience worse health outcomes. Evidence was strong for an association of LHL with smoking and having a suboptimal transplantation process;
- the few available HL-tailored interventions mainly used web-based strategies to inform and educate CKD patients. These interventions gave weak evidence that they improved knowledge, decision-making and health behaviours in CKD patients with LHL; and
- considerable research gaps remain. There are limited studies in earlier stages of CKD and thus on chances for prevention of progression towards severe kidney disease in patients with LHL. In addition, studies that unravel the role of the healthcare professionals in the support of LHL patients are lacking.

**What impact this may have on practice and policy?**

- healthcare organizations should improve the support of patients with LHL to prevent worse health outcomes. Although the best intervention strategies remain underexplored, web-based education was promising for improving patients’ knowledge and behaviours. Organizations could best start by implementing strategies that target smoking and the transplantation process;
- especially in earlier stages of CKD, more research is needed to unravel the mechanisms by which LHL leads to worse health outcomes. Additionally, research needs to develop and assess the effectiveness of HL-tailored interventions to improve these outcomes; and
- this should lead to further unravelling of LHL-associated mediating factors and enable targeting them with health literacy interventions, especially in earlier stages of CKD, to slow down and prevent the global rise of kidney disease.

## INTRODUCTION

In the last few decades, the number of people suffering from chronic kidney disease (CKD) has steadily increased [1, 2]. In the USA, people between the ages 30 and 49 years have a 54% chance of experiencing CKD during the course of their lives [3]. Often, kidney deterioration is almost unnoticeable, potentially leading to end-stage kidney disease, which is associated with high morbidity, mortality and economic burden [3]. The growing prevalence of CKD indicates a need to prioritize the development of interventions to retard or prevent this disease [4].

About 25% of CKD patients experience limited health literacy (LHL) [5]; this has been shown to be associated with worse health outcomes [6], such as faster kidney deterioration [7, 8] and higher mortality [9]. Health literacy (HL) is defined as the degree to which individuals have the capacity to obtain, process and understand basic health information and services needed to make appropriate health decisions [10]. Previous systematic reviews have summarized the evidence on predictors [5, 11] and serious negative impact of LHL in CKD [6, 11]. However, these did not address the available evidence on the mechanisms by which LHL leads to worse health outcomes and how interventions can target these mechanisms to improve that situation.

The Pathway of Paasche-Orlow provides a theory of patient- and system-level mechanisms which contain multiple factors that might mediate the relation between LHL and health outcomes [12]. Targeting these mediating factors with interventions potentially improves the health of patients with LHL. Patient-level factors refer to the patients’ capacities for self-management (e.g. medication adherence), utilization of care (e.g. seeking and obtaining professional help) and patient–provider (P–P) interaction (e.g. effective communication). However, these capacities highly depend on system factors, such as health system complexity, the patient’s social context and the capacities of the healthcare professional [12]. In other research fields, LHL has been found to be associated with several of these mediating factors [13–16], but the role of these factors in CKD is unclear.

The first research agenda on CKD and HL [17] and the European project Intervention Research On Health Literacy among Ageing population (IROHLA) recommend that, to prevent worse health outcomes, interventions should focus on both patients and professionals [18, 19]. State-of-the-art interventions should aim to inform and educate, teach skills, support behaviour change, strengthen social and professional support, and facilitate the involvement of individuals at a system level. Preferably, such interventions should be customized to the patient’s specific health context or environment [19]. Although in non-CKD care settings, HL-tailored interventions have been found to be effective in improving both patient [20–23] and professional [24] capacities, for CKD it remains uncertain how interventions can most effectively improve health outcomes of LHL patients.

This systematic review therefore aims to identify potential targets and strategies by summarizing the evidence on: (i) patient- and system-level factors that potentially mediate the relation between LHL and health outcomes; and (ii) the effectiveness of HL interventions that are customized to CKD patients.

## MATERIALS AND METHODS

We performed this systematic review in line with the principles of Preferred Reporting Items for Systematic Reviews and Meta-Analyses (PRISMA) [25].

### Search strategy and eligibility

Two reviewers (M.D.B. and E.M.F.) developed the search strategy and eligibility criteria with the support of two database search experts from the University Medical Center Groningen. After a pilot search to determine sensitivity and specificity, and discussion with a third reviewer (A.F.W.), the strategy was finalized. The search strategy aimed to retrieve original English, French or German peer-reviewed quantitative, qualitative and intervention studies related to HL and CKD. The final search strategy included a combination of CKD-specific terms, such as ‘chronic kidney’ or ‘dialysis’ and ‘renal transplant’ and HL related terms, such as ‘literacy’ and ‘numeracy’. Details on the search strategy are in Supplementary data, Table S1a–c.

Studies were eligible for inclusion if they: (i) included (a cohort of) any stage CKD patients aged ≥18 years and/or healthcare professionals; (ii) assessed HL using a validated screener or questionnaire; (iii) gave results on associations of LHL with potential mediating factors, derived from the Pathway of Paasche-Orlow; or (iv) provided information on the development and testing of interventions, customized to CKD and the needs of LHL patients. We excluded studies that: (i) used educational level as a measure of HL; (ii) focused solely on associations of HL with knowledge or health outcomes; or (iii) developed or validated HL screeners. Further information about the inclusion and exclusion criteria can be found in Supplementary data, Table S2.

### Study selection

Two reviewers (M.D.B. and E.M.F.) performed a systematic database search in Medline, Embase and Web of Science. They used an Excel file with main author, year and title to guide study selection. Both reviewers read titles and abstracts of all identified unique records to include studies that met the inclusion criteria. Disagreements were solved by discussion. If there was still uncertain about eligibility, then the reviewers read the full-text publication and decided based on a new discussion.

### Data extraction

The two reviewers then performed a full-text review of the included publications and filled in a data extraction table in Excel. Extracted data regarded study characteristics, study aims, main results and conclusions. For each study, data on associations between LHL and mediating factors were sorted into different columns in the Excel file, based on the mechanisms in the Pathway of Paasche-Orlow: self-care management, P–P interaction and utilization of care. The extraction file also encompassed columns to extract data on LHL and the role of the social context or competences of the healthcare professional, which came from IROHLA Intervention model [18]. Clinical health outcomes, such as kidney decline or blood pressure, were in a different column in the file. This structure helped to unravel the HL–mediators–health outcomes pathway. For intervention studies, we added to the table information about the chosen strategies and its effectiveness, also derived from IROHLA [18].

### Quality assessment

M.D.B. and E.M.F. rated the methodological quality of the included quantitative and intervention studies with the checklist of Downs and Black [26] and three additional criteria from the Effective Public Health Practice Project (EPHPP) Quality Assessment Tool and Appraisal Tool for Cross-sectional studies (AXIS) [27, 28]. Disagreements were solved in discussion with a third reviewer, A.F.W. The EPHPP and AXIS criteria were added to put more weight on potential participation bias, because of known lower research participation of people with LHL [29]. Qualitative studies were assessed with a checklist, derived from the Cochrane Supplemental Handbook Guidance [30].

Together, the tools provided 16 criteria for quantitative studies, 30 for intervention studies and 18 for qualitative studies within four domains: (i) reporting; (ii) external validity; (iii) internal validity; and (iv) study participation. Each criterion could be rated with 0, 1 or 2 points. The total rating for all criteria and each independent domain was expressed as a percentage of the total maximum score possible. Domains could be of low (≤50%), moderate (>50% and ≤75%) or high (>75%) quality. Both the total and domain ratings were used to determine the final study quality. A high-quality study had a total score >75% and at least three domains with a high-quality rating. Details on the rating system are in Supplementary File S3a.

### Evidence synthesis

Following the quality assessment, M.D.B. performed an evidence synthesis, which was checked by A.F.W. The synthesis aimed to determine the strength of evidence regarding an association of LHL with a specific mediating factor or, regarding the effectiveness of targeting a factor in interventions, based on number and quality of studies reporting results. This method of evidence synthesis is based on other publications [31, 32]. The synthesis led to three levels of strength of evidence for the existence of an association or effective intervention target; (i) strong: consistent findings in one high-quality study and at least two moderate-quality studies; (ii) moderate: consistent findings in at least three studies, of lower quality than (i); (iii) and weak: inconsistent findings irrespective of study quality or less than three studies available.

## RESULTS

**FIGURE 1:**
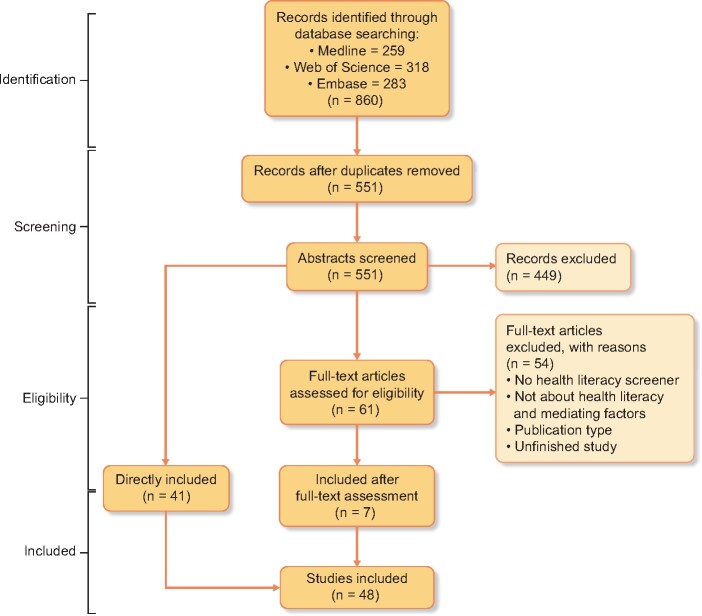
PRISMA flow diagram describing the search and record review process for this study.

Figure 1 shows the PRISMA diagram of our systematic review. The final search yielded 860 articles, written between 1987 and 2019. Forty-eight studies were eligible for inclusion. Main reasons for exclusion were: (i) used educational level as measurement for HL and (ii) study type.

### Study characteristics

**FIGURE 2:**
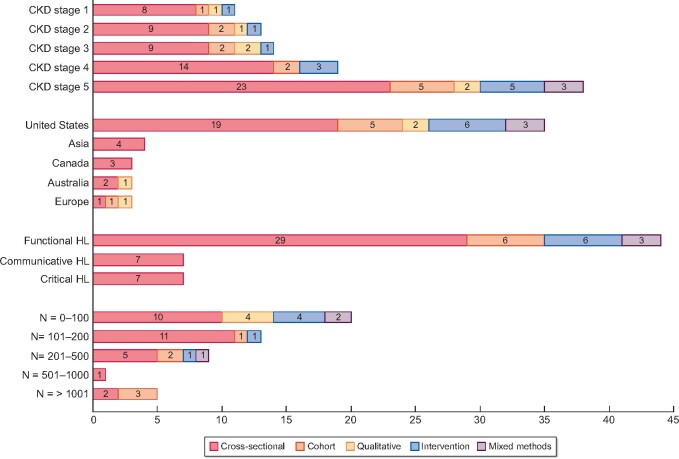
Overview of the characteristics of the included 48 studies: study population (CKD stage, region and number of participants) and measured domains of HL, specified by study type. The total numbers per category sometimes exceed 48 as some studies were counted multiple times because they addressed multiple CKD stages or multiple HL domains.

Figure 2 gives an overview of the main characteristics of the included studies. We identified 38 cross-sectional, cohort or mixed-method studies, 4 qualitative and 6 intervention studies, all in the English language. Most studies had sample sizes ˂200 (*n* = 33), were conducted in the USA (*n* = 35), and focused mainly on dialysis and transplant patients (*n* = 38). Only seven studies measured multiple HL domains, instead of just functional HL. Details on authors, year of publication, study population, sample size and used HL screener are in Tables 1–3.

**Table 1.** Study characteristics of descriptive quantitative studies and results on study quality and associations and findings within the HL–mediators–health outcomes pathway, organized by the Paasche-Orlow-derived mechanisms

Study characteristics				Study results			
Study	CKD-pop (*N*) country	Design	Measure (% LHL)	Q	Mechanism(s)	Association of health literacy with mediator within this mechanism(s) or other result related to mechanism	Association of health literacy or mediator with health outcome?
Studies with results on multiple mechanisms (*n* = 18)							
Devraj *et al*. [33]	1–4 (181) USA	Cross-sectional	NVS^a^ (63)	+	Self-care management Utilization of care	Yes: CKD awareness with self-management behaviours No: LHL with CKD awareness or duration of participation in clinic	Yes: Worse kidney function associated with higher CKD awareness No: LHL with severity of CKD
Taylor *et al.* [34]	5 (6842) UK	Cross-sectional	SILS^a^ (14.6)	+	Self-care management Utilization of care	Yes: LHL with current smoking Other: LHL is more prevalent in non-waitlisted incident dialysis (20%) patients than in waitlisted dialysis patients (15%) Transplant recipients have the lowest prevalence of LHL (12%)	Yes: LHL with more comorbidities, long-term disabilities, depression and psychosis
Ricardo *et al.* [8]	1–3 (2340) USA	Cross-sectional	sTOFHLA^a^ (16)	+	Self-care management Utilization of care	Yes: LHL with current smoking, perceived health and more frequent visits to the nephrologist No: LHL with medication use	Yes: LHL with lower eGFR, higher urine protein, more cardiovascular disease and more diabetes
Chen *et al.* [35]	1–5 (410) Taiwan	Cross-sectional	Mandarin HL scale^a^ (n.a.)	±	Self-care management Social context	Yes: LHL with worse self-management behaviours and decreased function of social support. Social support associated with self-management behaviours and treatment adherence	No results reported on health outcomes
Serper *et al.* [36]	5 (T) (98) USA	Mixed-method	NVS^a^ (37)	±	Self-care management Social context	Yes: LHL with choosing to spend money on expenses other than medication. These decisions were associated with lower medication adherence and explained by the social context	Yes: Choosing to spend money on expenses other than medications with higher rates of hospital admission
Demian *et al.* [37]	5 (T) (96) Canada	Cross-sectional	HL-Q^a,^^b,^^c^ (n.a.)	±	Self-care management Utilization of care P–P interaction	Other: Multifaceted HL screener indicates: actively managing health is the greatest HL challenge for transplant recipients, while navigating the health system, engaging with providers and understanding information are minor HL challenges	Yes: Appraising/understanding information associated with worse kidney health
Jain *et al.* [38]	5 (D) (32) USA	Cross-sectional	REALM^a^ (19)	±	Self-care management Utilization of care	No: LHL with treatment regimens, time on peritoneal dialysis or hospitalization	No: LHL with peritonitis, exit-site infections or dialysis adequacy
Kazley *et al.* [39]	5 (92) USA	Cross-sectional	REALM^a^ NVS^a^ (n.a.)	±	Utilization of care Social context	Yes: LHL with lower likelihood of being waitlisted for transplantation and lower social support	Yes: LHL with worse transplant outcomes
Lai *et al.* [40]	5 (D) (63) Singapore	Cross-sectional	FCCHL^a,^^b,^^c^ (n.a.)	±	Self-care management Utilization of care	Yes: LHL with worse blood glucose testing and foot care. Limited communicative and critical with worse diabetes self-management. Limited communicative HL with less exercise Limited critical HL associated with worse general diet No: LHL with duration of diabetes treatment	No: LHL with blood glucose levels
Gordon *et al.* [41]	5 (T) (124) USA	Cross-sectional	sTOFHLA^a^ (9) REALM^a^	±	Self-care management Utilization of care	Yes: LHL with shorter time after transplant. In open questions: patients express the need to improve understanding of transplantation and medication use	Yes: LHL with higher serum creatinine levels
Wright Nunes *et al*. [42]	1–4 (399) USA	Cross-sectional	REALM^a^ (n.a.)	±	Self-care management P–P interaction	Yes: LHL with lower perceived kidney disease specific knowledge No: LHL with satisfaction with the provider	Yes: Lower knowledge with lower eGFR awareness of CKD
Zhong *et al.* [43]	1–5 (61) USA	Cross-sectional	REALM^a^ (40.7)	−	Self-care management Utilization of care P–P interaction	Yes: LHL with medication and lifestyle behaviours, lower healthcare transition readiness from paediatric care to adult care services (a.o. ability to visit doctors and make appointments), less seeking of information and asking questions in a group of 18–29 years adolescents. Communication with providers positively influences knowledge. Greater nutrition knowledge predicted healthcare transition readiness	No results reported on health outcomes
Photharos *et al.* [44]	2–4 (275) Thailand	Cross-sectional	HLS-14^a^^,b,c^	−	Self-care management Social context	Yes: LHL influences self-efficacy in and performance of lifestyle activities. Self-efficacy is not a mediator of association between LHL and self-management No: LHL has no direct or indirect effect on social support or family functioning	No results reported on health outcomes
Dodson *et al.* [45]	5 (D) (913) Australia	Cross-sectional	HL-Q ^a,b,c^ (n.a.)	−	Self-care management Utilization of care P–P interaction Social context	Other: Multifaceted HL screener indicates: compared to a control group of other chronic patients, actively managing health is a greater HL challenge for dialysis patients, while they are better in navigating the health system, engaging with providers, understanding and applying information and enabling social support	Yes: LHL with worse serum albumin, depressive and anxiety symptoms and disease and mental burden
Patzer *et al.* [46]	5 (T) (99) USA	Mixed-method	REALM^a^ (24.7)	−	Self-care management Utilization of care	Yes: LHL with lower medication knowledge and self-reported treatment adherence No: LHL with demonstrated proper use of medications and hospitalization	No: LHL with graft rejection
Tuot *et al.* [47]	1–5 (264) USA	Cross-sectional	Brief HLS^a^ (46.6)	−	Self-care management P–P interaction	Yes: Providers’ word choice important to create awareness about CKD No: LHL with CKD awareness	No results reported on health outcomes
Lambert *et al*. [48]	4–5 (153) Australia	Cross-sectional	HeLMS^a^^,b,c^ (n.a.)	−	Self-care management Utilization of care P–P interaction	Other: Multifaceted HL screener indicates: incorporation of lifestyle is the greatest HL challenge. Filling in forms and accessing healthcare is a frequent HL problem. Communication with providers is a greater HL challenge for peritoneal dialysis patients compared with other CKD patients	No results reported on health outcomes
Dageforde *et al.* [49]	5 (104) USA	Cross-sectional	Brief HLS^a^ (23.1)	−	Utilization of care P–P interaction	Yes: LHL with not knowing the next step in the transplantation process. Attending consultations improves transplant knowledge and gives more concerns about finding a donor No: LHL with first-time centre visits	No results reported on health outcomes
Studies with results on self-care management (*n* = 9)							
Schrauben *et al.* [50]	1–3 (5499) USA	Cohort study	sTOFHLA^a^ (13)	+	Self-care management	Yes: LHL with less healthy behaviour patterns (smoking, obesity, lack of physical activity etc.) in ≥65 subgroup	Yes: Less healthy patterns associated with increased risk of dead, CKD progression and cardiovascular risks
Wong *et al.* [51]	1–4 (137) USA	Cross-sectional	HL-Q^a^^,b,c^ (26)	+	Self-care management	Yes: LHL with decreased fast food intake No: LHL with medication adherence and physical activity	No results reported on health outcomes
Devraj *et al*. [7]	1–4 (150) USA	Cross-sectional	NVS^a^ (63)	+	Self-care management	Yes: LHL with decreased self-management knowledge and decreased controlling for blood pressure No: LHL with other self-management knowledge, such as taking medication, sugar and salt intake, having lab checks	Yes: LHL with lower eGFR
Eneanya *et al.* [52]	4–5 (149) USA	Cross-sectional	REALM^a^ (34)	−	Self-care management	Yes: LHL with reduced knowledge of cardiopulmonary resuscitation. LHL mediates racial disparities for CPR knowledge	No results reported on health outcomes
Jones *et al.* [53]	4–5 (D) (41) Canada	Cross-sectional	sTOFHLA^a^ (5)	−	Self-care management	Yes: LHL with lower transplant and medication knowledge, lower adherence confidence, higher beliefs in medication importance and concerns regarding side effects	No results reported on health outcomes
Umeukeje *et al.* [54]	5 (D) (100) USA	Cross-sectional	sTOFHLA^a^ (50)	−	Self-care management	No: LHL with self-motivation of dialysis patients to adhere to phosphate treatment	Yes: Lower self-motivation and medication adherence with lower serum phosphorus levels
Adeseun *et al.* [55]	5 (D) (72) USA	Cross-sectional	sTOFHLA^a^ (21)	−	Self-care management	No: LHL with history of tobacco use	Yes: LHL with higher blood pressure No: LHL with other lifestyle markers, such as BMI
Green *et al.* [56]	5 (D) (288) USA	Cohort study	REALM^a^ (16)	−	Self-care management	No: LHL with quality of life	Yes: LHL with burden of comorbidities No: LHL with symptom burden, depression, dialysis adequacy and lab values (i.e. albumin, haemoglobin)
Foster *et al.* [57]	5 (D) (62) USA	Cross-sectional	sTOFHLA^a^ (30.3)	−	Self-care management	No: LHL with disaster preparedness (such as having extra medications)	No results reported on health outcomes
Studies with results on mechanisms related to utilization of care (*n* = 10)							
Taylor *et al.* [58]	5 (D) (2274) UK	Cohort study	SILS^a^ (24)	+	Utilization of care	Yes: LHL with reduced access to deceased-donor transplant listing and receiving a transplant from a living donor. This is likely related to patients’ preparation No: LHL with pre-emptive waitlisting or dialysis modality	No: LHL with catheter use or mortality
Warsame *et al.* [59]	4–5 (D) (1578) USA	Cohort study	Brief HLS^a^ (8.9)	+	Utilization of care	Yes: LHL with lower likelihood of being waitlisted for kidney transplant	Yes: LHL with lower likelihood of undergoing living donor transplant and greater risk of waitlist mortality
Green *et al.* [60]	5 (D) (260) USA	Cohort study	REALM^a^ (16)	+	Utilization of care	Yes: LHL with missed dialysis treatments, more emergency department visits, and more hospitalization No: LHL with abbreviating dialysis treatments	Yes: LHL with higher prevalence of comorbidities and fistula use No: LHL with mortality, lab values or receiving transplant
Dageforde *et al.* [61]	5 (T) (360) USA	Cross-sectional	SLS^a^ (10)	±	Utilization of care	Other: LHL more prevalent in patients with a deceased donor (14%) than in patients with a living donor (9%). Living donors have even lower prevalence of LHL (6%)	No results reported on health outcomes
Levine *et al*. [62]	2–5 (142) USA	Cohort study	NVS^a^ (12)	−	Utilization of care	No: LHL with emergency department visits, hospitalization or length of hospital stay	No results reported on health outcomes
Vilme *et al.* [63]	4–5 (D) (155) USA	Cross-sectional	REALM^a^ REALM-sf^a^ (n.a)	−	Utilization of care	No: LHL with patient interest in receiving a kidney from a living donor or with facilitators or barriers to pursue a living donor kidney transplantation, in a cohort of African-Americans	No results reported on health outcomes
Wong *et al.* [64]	4–5 (121) Canada	Cross-sectional	SLS^a^ (n.a.)	−	Utilization of care	Yes: LHL with requiring help to fill in measurements with tablets, and finding this task difficult or tiring	No results reported on health outcomes
Flythe *et al.* [65]	4–5 (154) USA	Cross-sectional	REALM^a^ (43.3)	−	Utilization of care	Yes: LHL shows a trend towards higher likelihood of 30-day hospital readmission (non-significant in adjusted models)	No results reported on health outcomes
Tohme *et al.* [66]	5 (D) (286) USA	Mixed-method	REALM^a^ (16)	−	Utilization of care	Yes: LHL with missing dialysis No: LHL with patients’ abbreviation of dialysis treatment	Missing dialysis with mortality. Abbreviation with hospitalization
Grubbs *et al.* [67]	5 (D) (62) USA	Cross-sectional	sTOFHLA^a^ (32.3)	−	Utilization of care	Yes: LHL with lower referral change for transplant evaluation No: LHL with treatment preference, uncertainties about treatment decision or being waitlisted	No results reported on health outcomes
Studies with results on mechanisms related to P–P interaction (*n* = 1)							
Bahadori *et al.* [68]	5 (D) (130) Iran	Cross-sectional	HELIA^a^^,b,c^ (53.8)	−	P–P interaction	Yes: Various subdomains of LHL (understanding and using information, decision-making) with perceived general health	Yes: LHL with physical and psychological symptoms

CKD-pop: population of interest by CKD stages (1, 2, 3, 4 or 5), when applicable specified for transplant (T) or dialysis (D); NVS: Newest Vital Sign; SILS, Single Item Literacy Screener; sTOFHLA: short Test of Functional Health Literacy in Adults; eGFR, estimated glomerular filtration rate; Mandarin HL Scale, Mandarin HL Scale; HL-Q, Health Literacy Questionnaire; REALM-SF, Rapid Estimate of Adult Literacy in Medicine—Short Form; FCCHL, Functional Communicative Critical Health Literacy; HLS, Health Literacy Scale; HeLMS, Health Literacy Management Scale; SLS, Short Literacy Survey; HELIA, Health Literacy for Iranian Adults; BMI, body mass index; n.a., not available; *N*, number of participants in the study; Q, study quality; +, high-quality study; ±, moderate-quality study; −, low-quality study, based on quality assessment.

a Functional HL measure.

b Communicative HL measure.

c Critical HL measure.

**Table 2.** Study characteristics of qualitative studies and results within the HL–mediators–health outcomes pathway

Study characteristics				Study results		
Study	CKD-pop (*N*) country	Design	Measure (% LHL)	Q	Mechanism(s)	Main results
Ladin *et al.* [69]	5 (D) (31) USA	Semi-structured interviews	–	+	Self-care management P–P interaction Social context	Decision-making is influenced by the patients’ lack of knowledge or skills. Providers use too difficult words and providers’ knowledge superiority limits shared decision-making. Providers also lack competences and time to discuss end of life care preferences. Patients consider the support system too emotional to discuss end of life care and speaking to other patients helpful to facilitate decision-making
Van Dipten *et al.* [70]	1–3 (25) The Netherlands	Semi-structured interviews	–	±	Self-care management P–P interaction	Patients mention reasons for self-management problems, such as knowledge gaps and misconceptions, absence of symptoms, reduced sense of seriousness and problems with linking lifestyle to disease risks. Provider attitudes in earlier stages of CKD create this reduced sense of seriousness. Patients also feel providers lack time and energy to tailor information to their needs and to explain details
Sakraida and Robinson [71]	3 (6) USA	Focus group discussion	–	±	Self-care management P–P interaction	Patients mention knowledge gaps as barrier to effective self-management, and the need for encouraging messages to improve self-management. Patients mention to searching for information online but being uncertain about quality and source of information. Patients mention providers as their main source of information. They prefer face-to-face contact with simple information and perceive their own lack of assertiveness and provider-oriented care plans as barriers in consultations
Muscat *et al.* [72]	5 (D) (35) Australia	Semi-structured interviews	–	−	Self-care management P–P interaction Social context	Patients believe their lack of awareness and knowledge, paternalistic styles of providers and time are barriers in decision-making. Patients often expect professionals to decide. Patients regard information as important to know what to expect, but not necessarily to inform decision-making. They also mention that communication with general practitioners is easier than with specialists. Patients also mention that family influences the process of decision-making

CKD-pop, population of interest by CKD stages (1, 2, 3, 4 or 5), when applicable specified for transplant (T) or dialysis (D); *N,* number of participants in the study; Q, study quality; +, high-quality study; ±, moderate-quality study; −, low-quality study, based on quality assessment.

**Table 3.** Study characteristics of intervention studies and target mechanisms, objectives, study approaches and main results of included intervention studies

Study	CKD-pop (N) Country	Design	Measure (%LHL)	Q	Target mechanism	Intervention objectives and target group	Approach of intervention	Main results
Patzer *et al.* [73]	4–5 (D) (470) USA	RCT study	NVS^a^ (20.3)	±	Utilization of care P–P interaction	Inform and educate patients Facilitate patient involvement Customize to context Strengthen professional support	iChoose kidney: a shared patient/provider web-based decision aid to provide individualized risk estimates of mortality and survival for different transplant and dialysis treatment options. Providers enter patient characteristics in the aid; outcome discussed during consultation	+ transplant knowledge* + access to transplantation + providers report improved discussion and patient knowledge − decisional conflict and preferences
Chandar *et al.* [74]	5 (T) (16) USA	Pre-post study	REALM-teen^a^ (43.8)	−	Self-care management	Inform and educate patients Support patient behaviour change	App with short quizzes and videos to improve knowledge about transplantation, medications, laboratory tests and care for transplanted kidneys. Also asked questions about patients’ health and medication adherence, to provide personal advice to support behaviour change	+ knowledge of names and purpose of medications* + satisfaction + feelings of empowerment
Timmerman *et al.* [75]	1–3 (21) USA	Pilot study	NVS^a^ (63)	−	Self-care management Social context	Inform and educate patients Teach skills to patients Support patient behaviour change Strengthen social support	6-week group intervention for patients on health literacy, quality of life, diet and self-efficacy, based on model of Health Promotion and designed to facilitate health-promoting behaviours. Each patient formulated personal goals to support problem solving	+ quality of life* + energy level* + health literacy* + dietary self-efficacy* + type of foods
Axelrod *et al.* [76]	4–5 (D) (81) USA	Pilot-study with focus groups	Adapted Brief HLS^a^ (40–73)	−	Utilization of care P–P interaction Social context	Inform and educate patients Facilitate patient involvement Customize to context Strengthen social support	My Transplant Coach, app where patients can enter essential demographic and clinical information. The app generates estimates of prognosis and uses videos to explain different transplant and dialysis treatment options. Facilitates easy sharing with professional	+ lower acceptability of app in patients with limited Internet experience* + benefits for all literacy levels* + confidence in conversation + transplant knowledge* + informed decision-making*
Robinson *et al.* [77]	5 (T) (170) USA	Pilot RCT study	sTOFHLA^a^ (28)	−	Self-care management Social context	Inform and educate patients Support behaviour change of patients Customize to context	SunProtect, digital education on personal skin cancer risk and sun-protection actions for patients, to use in the hospital. Information offered with videos, spoken language (English and Spanish) and culture-sensitive patient stories	+ sun-protection knowledge + awareness* + sun-protection use* + better results in patients with LHL*
Ameling *et al.* [78]	4–5 (D) (48) USA	Mixed- method	REALM^a^ (18)	−	Utilization of care Social context	Inform and educate patients Facilitate patient involvement Customize to context	Video and handbook for patients. Subjective and evidence-based information about positive and negative features of different treatment options to support patients and family in decision-making	+ refined content, based on feedback + comprehension of the aid + satisfaction + quality of the aid

CKD-pop, population of interest by CKD stage (1, 2, 3, 4 or 5), when applicable specified for transplant (T) or dialysis (D); RCT, randomized controlled trial; NVS, Newest Vital Sign; REALM-SF, Rapid Estimate of Adult Literacy in Medicine—Short Form; Brief HLS, Brief Health Literacy screener; sTOFHLA, short Test of Functional Health Literacy in Adults; *N*, number of participants in the study; Q, study quality; +, high-quality study; ±, moderate-quality study; −, low-quality study, based on quality assessment. *Significant effect (P ≤ 0.05).

a Functional HL measure.

### Quality assessment

Nine quantitative studies and one qualitative study were of high quality. Nine quantitative studies, two qualitative studies and one intervention study were of moderate quality. The other 26 studies were of low quality. The risk for external validity bias was high: only two studies could fully ascertain the study population was a good representation of the total population. In 25 studies, participation bias was a risk: sample sizes were often not justified or participation rates were low. Within the domains reporting and internal validity, two criteria commonly caused risks of bias: (i) limited adjustment for confounders and (ii) not reporting actual probability values (e.g. 0.035 rather than 0.05). In qualitative studies, bias risks were often a consequence of inappropriate methodology: studies did for example not justify sampling procedure and data saturation. Most intervention studies used weak non-randomized control study designs, which led to low-quality ratings. Tables 1–3 show the overall quality rating and Supplementary data, Table S3b–d provide details on the domain ratings for each study.

### Strength of evidence for mediating factors

Table 1 summarizes the results on associations between LHL, patient- and system-level factors and health outcomes. In general, evidence was weak. Twenty-seven studies provided evidence for an association of LHL with potential mediating factors. Evidence was only strong for an association with smoking [7, 34, 50] and having a suboptimal transplantation process [39, 49, 58, 59, 67]. No studies explicitly assessed mediation. However, four studies provided weak evidence for a potential mediating role of factors related to self-care management [33, 36, 42] and utilization of care [66], finding independent associations with both LHL and health outcomes. Eleven studies found no associations of LHL with the factors of their interest. Details are in the following paragraphs.

#### Self-care management

Twenty-five studies gave generally weak evidence for an association of LHL with a variety of mediating factors related to self-care management. We found strong evidence for an association of LHL and current smoking [7, 34, 50]. Three studies provided weak evidence for mediation, finding associations of LHL with worse perceived CKD treatment knowledge [42], less healthy lifestyle patterns [33] and choosing to spend money on expenses other than medications [36], and, additionally, associations of these mediating factors with health outcomes. For other factors, evidence was weak or inconsistent. LHL was associated with worse control of blood pressure [55, 61], lower medication adherence confidence [44, 53] and lower quality of life [56]. For worse treatment and self-care knowledge [7, 41–43, 49, 52, 53, 79], worse self-care behaviours [7, 40, 43, 51, 55], including lifestyle [40, 50, 51] and adherence problems [8, 38, 46, 51, 53] some studies found an association with LHL, while others did not. LHL was not associated with CKD awareness [33, 47], treatment preferences [67], disaster preparedness [57] and phosphate regulation [54]. According to multidomain screeners, patients perceived self-care management as their biggest HL challenge, especially in severe CKD stages [37, 45, 80].

#### Utilization of care

Twenty-three studies provided generally weak evidence for an association of LHL with factors related to utilization of care. We found strong evidence that LHL is associated with a suboptimal transplant process, illustrated by a lower likelihood of being wait-listed for [39, 58, 59], or referred to [67] transplantation and ‘not knowing the next step in the transplant process’ [49]. Connected to this, we found weak evidence that LHL is more prevalent in non-waitlisted and deceased donor patients compared with waitlisted and living donor patients [34, 61]. For other transplant factors, such as treatment preference [63, 67] or attending evaluations [49], no HL associations were found. Furthermore, we found weak evidence that LHL was associated with visiting the nephrologist more often [8], problems using digital health information [64] and missing dialysis [60, 66]. For associations of LHL with higher rates of hospitalization [38, 46, 60, 62, 65] and more emergency department visits [60, 62] studies confirmed and denied HL associations. LHL was not associated with abbreviating dialysis [60, 66]. According to multidomain HL screeners, patients did not perceive utilization of care as a major challenge [37, 45, 48].

#### P–P interaction

Eight studies gave weak evidence on factors related to P–P interaction. CKD patients did not perceive engaging with providers as their greatest HL problem [37, 42, 45]. However, in adolescents ˃18 years, one study showed that LHL was associated with several behaviours related to communication [43]. Another study showed an association of these behaviours with perceived general health [68]. Healthcare professional visits [49] and simple word choice [47] positively influenced CKD awareness and knowledge. LHL was not associated with provider satisfaction [42].

#### Other system factors

Five studies provided weak evidence on associations of LHL with the social context. For an association of LHL with reduced social support evidence was weak [35, 39, 44]. The social context was a strong and independent factor influencing self-management behaviours [35] and medication trade-offs [36]. There was no evidence regarding other Paasche-Orlow-derived mechanisms, such as the HL competences of professionals.

#### Suggestions for intervention targets

Table 2 provides an overview of the four qualitative studies, which offer suggestions for intervention targets within different Paasche-Orlow-derived mechanisms. Patients indicated that a lack of knowledge [69–71] and symptoms [70], perceived disease seriousness [70] and struggles to find information [71] influence self-care management in earlier CKD stages. A lack of knowledge [69, 71, 72] and time [70, 72], perceived hierarchy [72], difficult language [69, 71] and insufficient information [70–72] were barriers for effective P–P interaction and treatment decision-making. To improve that situation, patients suggested easier language [69–71], peer support [69] and the role of social support [69, 71].

### Intervention effectiveness and strategies

Table 3 summarizes the approach and main results of the six included intervention studies, of which five were led in dialysis or transplant patients. Since the study quality was often low, we only retrieved weak evidence for intervention effectiveness. The interventions targeted multiple mediating factors, and were able to improve knowledge [73, 74, 76, 81], decision-making [73, 76] and self-care behaviours [75, 81], also specifically in patients with LHL [73, 75, 77].

The interventions mainly used digital, visual strategies [73, 76–78] and targeted patients. Specific interventions targeting professionals were absent. The interventions aimed to educate and teach skills [73–78], especially to support treatment decision-making [73, 75, 76, 78]. Two interventions on lifestyle [75] and sun-cancer protection [77] also aimed to support behavioural change. One study showed that implementation of a decision-making tool into consultations also strengthened professional support [73]. Several interventions had strategies of customization to the context, for example, by adapting the content to individual clinical information [73, 76] or cultural background [77]. Co-development by patients and professionals proved effective in improving comprehensibility, content and satisfaction with the interventions [74, 76, 78].

## DISCUSSION

Evidence on patient- and system-level mediating factors and effectiveness of interventions is generally weak, which impairs the identification of promising intervention targets and strategies. We found strong evidence for an association of LHL with a suboptimal transplant process and smoking. We retrieved only weak evidence for a variety of other factors that potentially mediate the relation between LHL and health outcomes. Moreover, we found weak evidence that HL-tailored intervention strategies were effective in improving knowledge, decision-making and health behaviours.

We retrieved strong evidence for an association of LHL with having a suboptimal transplant process [39, 49, 58, 59, 67] and smoking [7, 34, 50]. Since both factors relate to behaviours that have a negative effect on health outcomes in the general CKD population [81, 82], we consider them important targets for interventions. Our review adds LHL as an important factor negatively influencing the chance to receive a kidney transplant, next to the patients’ knowledge and beliefs, which were known to cause disparities in transplant access [83]. Our findings also support the results in other organ transplant settings that patients with LHL use care differently. For example, they need more emergency care [84], make less use of preventive services [85] and miss follow-up appointments more often [86]. Our review also strengthens the evidence from non-CKD studies, which show that LHL is associated with current smoking [87, 88], less knowledge about smoking, lower risk perceptions [89] and difficulties in stopping smoking [90]. In the general CKD population, patients are often unaware that smoking is a risk factor for kidney deterioration [91]. Our findings suggest that patients with LHL have reduced knowledge or lower ability to change behaviour. Customized interventions, particularly to support the transplantation process and stopping smoking, are needed to improve the outcomes of patients with LHL.

We found only weak evidence for a variety of factors that potentially mediate the relation between LHL and health outcomes. This impedes the drawing of strong conclusions on targets for interventions in CKD. Even though studies on HL in other diseases like diabetes and cardiovascular disease showed strong associations of LHL with mediating factors, such as knowledge, P–P communication, medication adherence and self-care behaviours [92, 93]. A potential explanation for our weak evidence could lie in our separate assessment of various mediating factors, instead of lumping them together. For example, we found separate associations of LHL with knowledge of medication [44, 46], lifestyle [7], disease [42], transplant [44, 49] and cardiopulmonary resuscitation [52]. We think that these factors are too heterogeneous to combine validly. However, one could argue that these studies together offer strong evidence for an association of LHL with knowledge. CKD studies should further examine the role of mediation in high-quality studies to unravel the mechanisms leading from LHL to health outcomes.

In agreement with HL interventions in other populations [20, 23, 94] and general CKD educational interventions [95, 96], our review gave weak evidence that CKD HL interventions were effective to improve knowledge [73, 74, 76, 81], decision-making [73, 76] and self-care behaviours [75, 81]. However, the included six interventions were unable to detect long-term behaviour change and an effect on health outcomes, and mostly used online or digital intervention strategies. Since patients with LHL also have more problems with technology [97], the effectiveness of the current strategies remains questionable. Research in other populations concludes multi-component interventions are the most successful to support people with LHL and emphasizes the importance of aiming at the health system [23, 98]. Our included qualitative studies [69–72], in which patients explicitly requested easier, non-medical language in consultations and inclusion of the social network, indicate other promising intervention strategies. Healthcare organizations and researchers should therefore develop and test a broader range of CKD interventions, targeting both patients and the health system, to bridge the barriers of LHL patients.

We identified several important research gaps. Most studies focused on dialysis and transplant patients. There is very little evidence on the improvement of outcomes of LHL patients in earlier stages of CKD, and thus on the prevention of progression towards severe kidney disease. Moreover, most studies are from the USA. The results from these studies should be confirmed for other parts of the world, as findings may be influenced by culture and specific characteristics of the health system. Finally, interventions that target the capacities of healthcare professionals are totally lacking.

Our review is, to our knowledge, the first to unravel associations of LHL in CKD with a specific intervention focus; previous reviews have instead focused on predictors and prevalence of LHL in CKD [5, 11] or on associations of LHL with outcomes [6, 11]. Our review has a number of strengths. The first strength is our inclusion of several study designs to provide a complete overview of potential intervention targets and strategies. The second is its comprehensive search strategy, used to search three databases. The study selection, data extraction and quality assessment were set up and reported according to PRISMA guidelines. A third strength is our use of the Pathway of Paasche-Orlow and the IROHLA model, offering a theory-based approach to summarize the evidence and to identify research gaps.

This review also has limitations. The first is our use of two different quality assessment tools, possibly resulting in differences in quality rating between quantitative and qualitative studies. However, because we used a strict classification system to increase comparability, we expect no major biases. The second limitation is that we did not ask for grey literature and excluded several study types. We therefore might have missed information, but since we still provide an extensive overview, it is our opinion that such additional evidence would not greatly affect our conclusions. The third limitation is that we could not assess the effects of the way of measuring HL. Most studies measured functional HL, the ability to read and understand written and oral health information. Broader definitions and measures of HL, which include communication and critical literacy and contextual factors, have become more common only recently. The used measure may affect the associations found with mediating factors, for which we could not account.

Our findings imply that healthcare organizations need to take action. Although the best intervention strategies remain underexplored, organizations could best start with targeting smoking behaviour and transplantation processes. The web-based strategies that we identified are promising for improving knowledge and decision-making, and need further implementation in healthcare settings. Additionally new strategies need to be developed. Policy makers should seek ways to simplify navigation in the health system to improve care access.

We found high-quality studies to be scarce. This shows a need for larger cohort and intervention studies to unravel the mechanisms by which LHL leads to worse health outcomes and to assess the effectiveness of HL-tailored interventions to improve these outcomes. Such research should include studies on earlier stages of CKD in various parts of the world to find ways to prevent kidney deterioration among people with LHL. Additionally, research is needed to adapt the activities of healthcare organizations to the needs of patients with LHL, for example, by strengthening the communication capacities of professionals. This may help to better inform patients with LHL and improve communication between these patients and professionals [99].

In conclusion, despite the call for urgency in the research agenda on CKD and HL in 2009 [17], effective intervention targets and strategies are still lacking. We urgently need funding agencies, policy makers, researchers and healthcare professionals to take the lead in efforts to improve the health outcomes of CKD patients with LHL. This should lead to unravelling of the mechanisms and targeting of LHL-associated mediating factors with HL interventions, especially in earlier stages of CKD, to slow down and prevent the global rise of kidney disease.

## SUPPLEMENTARY DATA

Supplementary data are available at ndt online.

## FUNDING

This research was conducted independently by the research team, but supported by funding from the Dutch Kidney Foundation. The research was funded by the Behavioural and Social Research Call, grant number: 17SWO06.

## AUTHORS’ CONTRIBUTIONS

M.D.B. and A.F.W. designed the study in line with PRISMA guidelines. The first search strategy was developed by E.M.F. and M.D.B. in close cooperation with the database search expert from the Department of Health Sciences and the database search expert from the Medical Library of the University Medical Center Groningen. M.D.B. and E.M.F. discussed the search strategy with A.F.W., and both E.M.F. and M.D.B. pilot-tested the strategy independently to determine specificity and sensitivity. After the first pilot-test, the search strategy was again discussed with A.F.W. and adapted, omitting terms related to ‘education’. After a second pilot-test, E.M.F., M.D.B. and A.F.W. decided that this would be the final search strategy. E.M.F. then developed a draft of an Excel file, which was checked and adapted by M.D.B. and then discussed with A.F.W. Using this Excel file, E.M.F. and M.D.B. independently screened title and abstract for all results. They discussed disagreements, and when still uncertain, consulted with A.F.W., E.M.F. and M.D.B. together developed a second Excel file for data extraction, which was discussed and adapted with A.F.W., E.M.F. and M.D.B. again performed an independent data extraction and discussed results to check for disagreements. M.D.B. performed the quality assessment and evidence synthesis, which were checked by E.M.F. or A.F.W. M.D.B. set up drafts of the article, which were discussed four times with A.F.W., S.A.R., G.N. and R.W. All authors added comments to the publication for each of the four discussion moments and did in-text suggestions for improvement. After consent from all authors above, the final publication was submitted by M.D.B. The results presented in this article have not been published previously in whole or part, except in abstract form.

## CONFLICT OF INTEREST STATEMENT 

None declared.

(See related article by Lameire and Vanholder. Health literacy problems of kidney patients. *Nephrol Dial Transplant* 2021; 36: 1155–1157)

## Supplementary Material

gfaa273_Supplementary_Data
