# Supplementary material for: How to tackle health literacy problems in chronic kidney disease patients? A systematic review to identify promising intervention targets and strategies
Source: Nephrol Dial Transplant. 2020 Dec 22;36(7):1207–21. doi: 10.1093/ndt/gfaa273 (PMC8237988; doi:10.1093/ndt/gfaa273)
Supplement: gfaa273_Supplementary_Data [file gfaa273_supplementary_data.zip › NDT-00476-2020 without tracks Supplemental Files 1-3a.docx]

**Table of contents: Additional Files**

[**Supplemental Tables 1a-1c: Search strategies for Pubmed, Web of Science and Embase**](#_Additional_file_1:)

[**Supplemental Table 2: Inclusion and exclusion criteria**](#_Additional_file_2:)

[**Supplemental Tables 3a: Quality**](#_Additional_file_3:_1) **assessment rating system**

## Supplemental Table 1a-1c: Search strategies

**Table 1a: Search strategy for PubMed**

| S1 | "Kidney Failure, Chronic"[Mesh] OR "Renal Insufficiency, Chronic"[Mesh] OR "Kidney Transplantation"[Mesh] OR "Renal Dialysis"[Mesh] OR chronic kidney[tiab] OR ckd[tiab] OR chronic renal[tiab] OR kidney transplant[tiab] OR renal transplant[tiab] OR dialysis[tiab] | - | 314132 |
| --- | --- | --- | --- |
| S2 | "Health Literacy"[Mesh] OR literacy[tiab] OR illiterate[tiab] OR illiteracy[tiab] OR literate[tiab] OR numeracy[tiab] OR TOFHLA[tiab]"Health Literacy"[Mesh] OR literacy[tiab] OR illiterate[tiab] OR illiteracy[tiab] OR literate[tiab] OR numeracy[tiab] OR TOFHLA[tiab] | - | 23876 |
| S3 | S1 AND S2 | - | 259 |

**Table 1b: Search strategy for Web of Science**

| S1 | TS=(literacy OR illiterate OR illiteracy OR literate OR numeracy OR TOFHLA) | - | 61467 |
| --- | --- | --- | --- |
| S2 | TS=("chronic kidney" OR ckd OR "chronic renal" OR "kidney transplant" OR "renal transplant" OR dialysis) | - | 232052 |
| S3 | S1 AND S2 | - | 317 |

**Table 1c: Search strategy for Embase**

| S1 | S1: (((((((((((chronic AND ('kidney' OR 'kidney'/exp OR kidney) AND ('failure' OR 'failure'/exp OR failure) OR chronic) AND ('kidney' OR 'kidney'/exp OR kidney) AND ('failure' OR 'failure'/exp OR failure) OR renal) AND insufficiency AND chronic OR renal) AND insufficiency AND chronic OR 'kidney' OR 'kidney'/exp OR kidney) AND ('transplant' OR 'transplant'/exp OR transplant) OR 'kidney' OR 'kidney'/exp OR kidney) AND ('transplant' OR 'transplant'/exp OR transplant) OR renal) AND ('dialysis' OR 'dialysis'/exp OR dialysis) OR renal) AND ('dialysis' OR 'dialysis'/exp OR dialysis) OR chronic) AND kidney:ab,ti OR ckd:ab,ti OR chronic) AND renal:ab,ti OR 'kidney' OR 'kidney'/exp OR kidney) AND transplant:ab,ti OR renal) AND transplant:ab,ti OR dialysis:ab,ti | - | 229419 |
| --- | --- | --- | --- |
| S2 | S2: 'health literacy' OR literacy:ab,ti OR illiterate:ab,ti OR illiteracy:ab,ti OR literate:ab,ti OR numeracy:ab,ti OR tofhla:ab,ti | - | 29283 |
| S3 | S1 and S2 | - | 282 |

## Supplemental Table 2: Inclusion and exclusion criteria

| **Inclusion criteria** | **Exclusion criteria** |
| --- | --- |
| Studies:   - Included (a cohort of) any stage CKD patients ≥18 and/or health care professionals. - Assessed HL using a validated HL screener or questionnaire. - Gave results on associations of LHL with potential mediating factors, derived from the Pathway of Paasche-Orlow or provided information on the development and testing of interventions explicitly customized to CKD and the needs of LHL patients. - Were original, peer-reviewed quantitative, qualitative and intervention studies in English, German or French. | Studies   - Only included patients <18 or with cognitive problems. - Only gave results about associations of HL with knowledge or HL with clinical health outcomes. - Described the development or validation of HL screeners or questionnaires, but without outcomes on factors potentially explaining worse health outcomes. - Used educational level (e. g. years at school) as measurement for HL. - Were incomplete or other study type: for example systematic reviews, congress abstracts, letters, descriptions. |

## Supplemental Table 3a: Quality assessment rating system

For each study, we conducted a quality assessment. MDB assessed the quality of the included studies, which was then checked by EMF or AFW. We assessed the quality of quantitative studies using the checklist of Downs and Black^26^. We added three items, derived from the Effective Public Health Practice Project Quality Assessment Tool,^27^ to detect potential validity problems related to study participation. We assessed the quality of qualitative studies using a checklist derived from the Cochrane Supplemental Handbook Guidance^28^. The following tables show the scores per study and the overall quality rating. We determined overall quality by examining both the total rating score and the quality in the separate domains. We chose this approach in order to facilitate comparison of the separate study types, where we used different checklists but similar assessment domains. In addition, we wanted to have a closer look at the domains of interest (reporting, external validity, internal validity, study participation) and weigh them in the overall quality assessment.

**Table 3a: Quality assessment rating system: combination of overall score and domain-specific quality rates determine study quality**

| **Quality** | **Score** | **Minimal demands related to separate quality domains** | | |
| --- | --- | --- | --- | --- |
| **High quality** | 14-17 (cc + cohort)  20-23 (qualitative)  26-31 (intervention) | **4 high quality** | **3 high quality**  1 moderate quality | **3 high quality**  1 low quality |
| **Moderate quality** | 12-14 (cc + cohort)  17-21 (qualitative)  22-26 (intervention) | **2 high quality**  2 moderate quality ­or 1 moderate and 1 low quality | **1 high quality** and 3 moderate quality | 4 moderate quality |
| **Low quality** | 0-13 (cc+cohort)  0-18 (qualitative)  0-23 (intervention) | **2 high quality**  2 low quality | All other combinations, with max. 1 high quality |  |

The ratings of the separate studies are in Supplemental File 3b-3d
