# Supplementary figures and images for: How to tackle health literacy problems in chronic kidney disease patients? A systematic review to identify promising intervention targets and strategies
Source: Nephrol Dial Transplant. 2020 Dec 22;36(7):1207–21. doi: 10.1093/ndt/gfaa273 (PMC8237988; doi:10.1093/ndt/gfaa273)

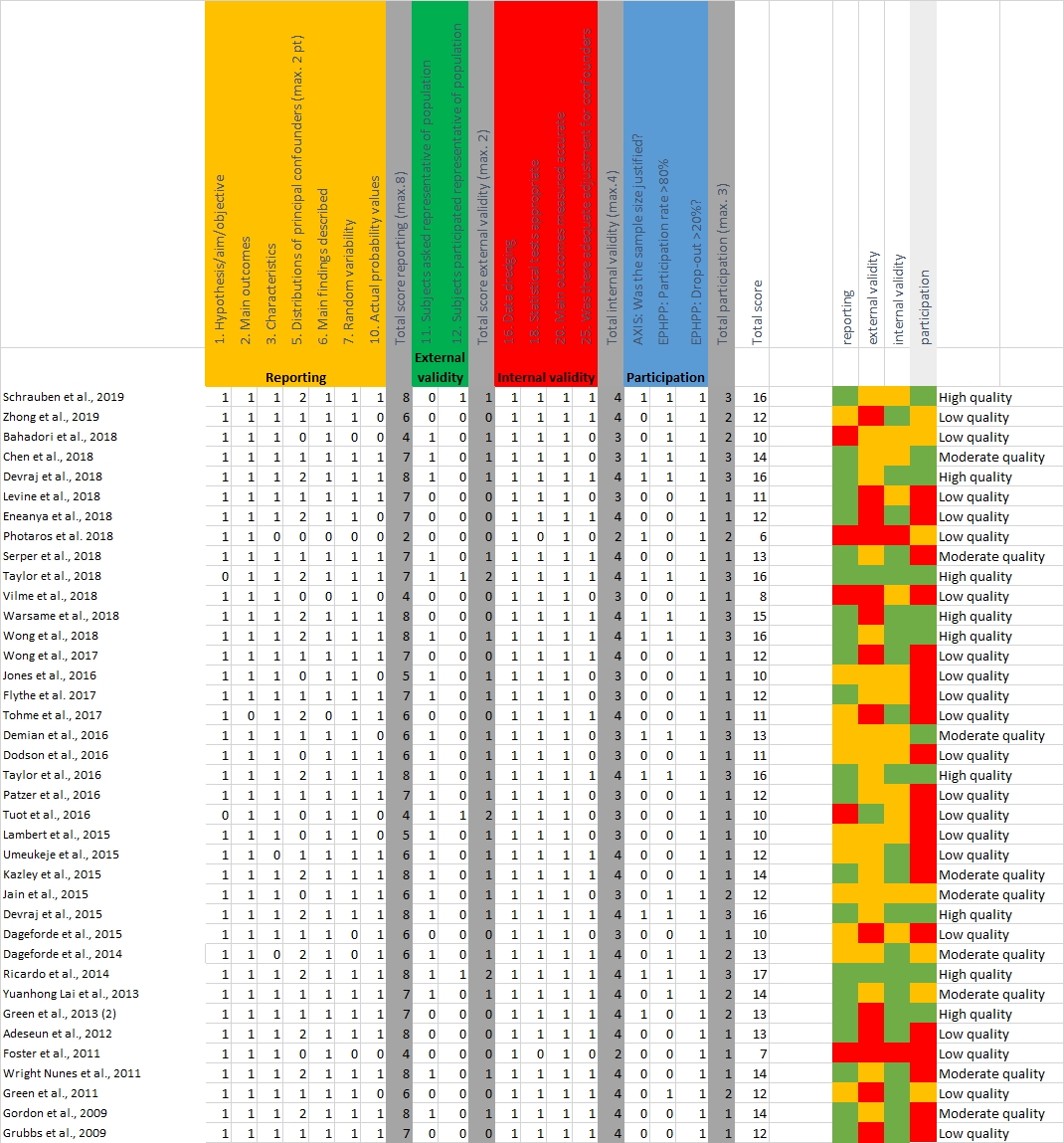

Supplement: gfaa273_Supplementary_Data [file gfaa273_supplementary_data.zip › MBo A1 Supplemental file 3b.jpg]

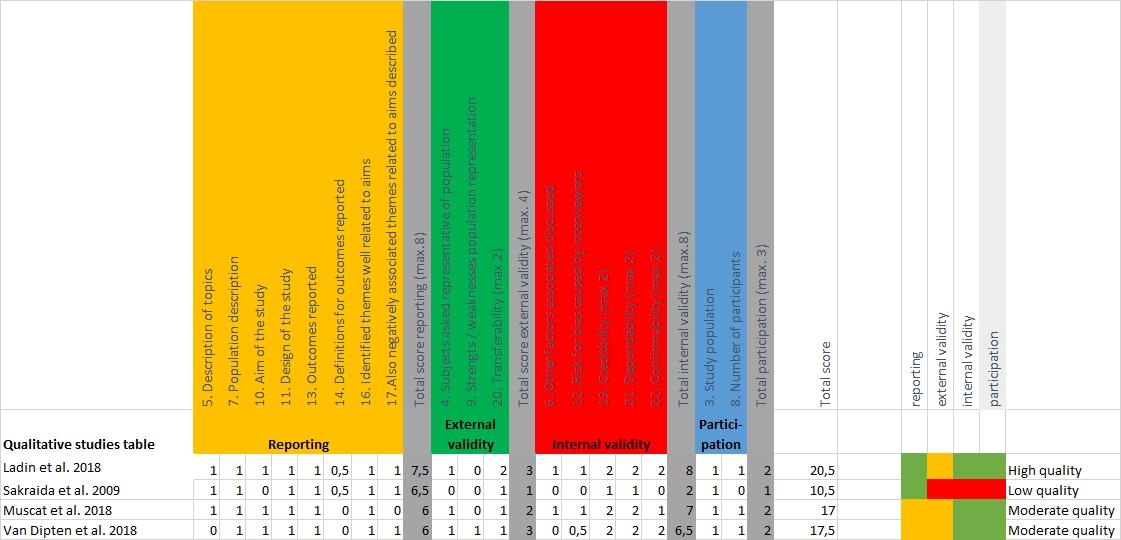

Supplement: gfaa273_Supplementary_Data [file gfaa273_supplementary_data.zip › MBo A1 Supplemental file 3c.jpg]

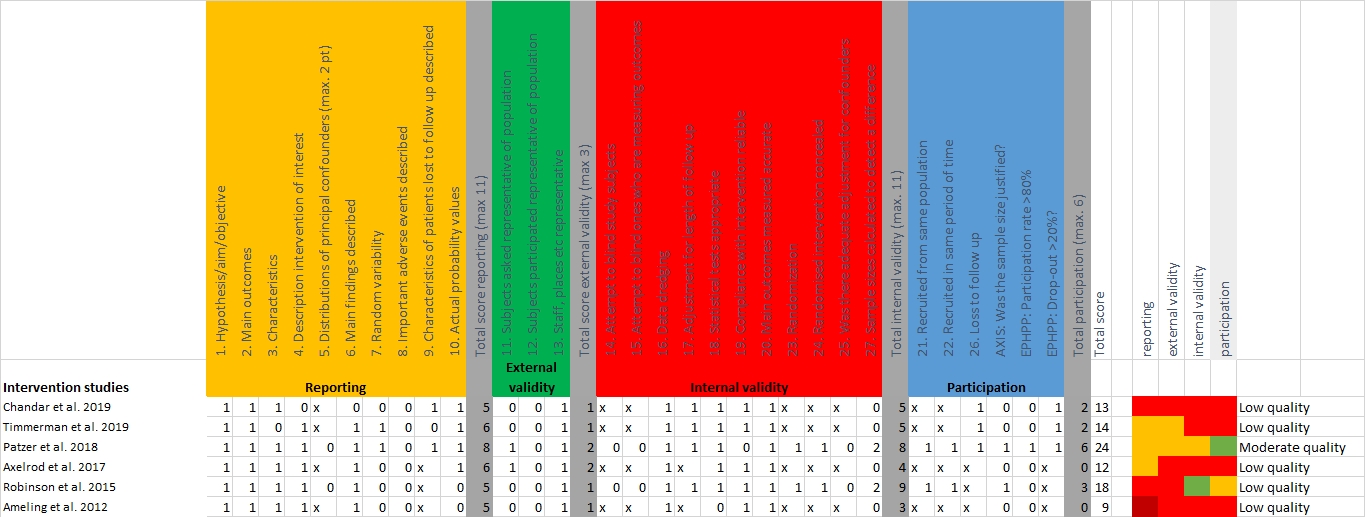

Supplement: gfaa273_Supplementary_Data [file gfaa273_supplementary_data.zip › MBo A1 Supplemental file 3d.jpg]
